# Exploring the Antitumor Mechanisms of Zingiberis Rhizoma Combined with Coptidis Rhizoma Using a Network Pharmacology Approach

**DOI:** 10.1155/2020/8887982

**Published:** 2020-12-24

**Authors:** Meng Wang, Youke Qi, Yongning Sun

**Affiliations:** ^1^Shanghai Jiao Tong University Affiliated Sixth People's Hospital, Shanghai 200233, China; ^2^Shanghai Municipal Hospital of Traditional Chinese Medicine, Shanghai University of Traditional Chinese Medicine, Shanghai 200071, China

## Abstract

**Background:**

Although the combination of Zingiberis rhizoma (ZR) and Coptidis rhizoma (CR) is a classic traditional Chinese medicine-based herbal pair used for its antitumor effect, the material basis and underlying mechanisms are unclear. Here, a network pharmacology approach was used to elucidate the antitumor mechanisms of ZR-CR.

**Materials and Methods:**

To predict the targets of ZR-CR in treating tumors, we constructed protein–protein interactions and hub component-target networks and performed pathway and process enrichment and molecular docking analysis. We used a surface plasmon resonance (SPR) assay to validate the predicted component-target affinities. Hub gene expression and survival analysis in patients with tumors were used to predict the clinical significance.

**Results:**

The active components of ZR-CR—shogaol, daucosterol, ginkgetin, berberine, quercetin, chlorogenic acid, and vanillic acid—exhibited antitumor activities via the MAPK, PI3K-AKT, TNF, FOXO, HIF-1, and VEGF signaling pathways. Molecular docking and SPR analyses suggested direct binding of berberine with AKT1 and TP53; quercetin with EGFR and VEGF165; and ginkgetin, isoginkgetin, and daucosterol with VEGF165 with weak affinities. Gene expression levels of the hub targets of ZR-CR were associated with overall survival and disease-free survival in patients with various tumor types.

**Conclusions:**

The antitumor components of the ZR-CR herbal pair and the mechanisms underlying their antitumor effects were identified. These antitumor components deserve to be explored further in experimental and clinical studies.

## 1. Introduction

Although considerable efforts have been made to improve tumor diagnosis and treatment, tumors are still a serious threat to human health. Tumors are recognized as an important cause of morbidity and mortality worldwide; in nearly 100 countries around the world, cancer is the first or second leading cause of premature death [1, 2].

Traditional Chinese medicine (TCM), as an effective treatment method, has been used to treat patients with various clinical diseases, including cancer. TCM has developed over thousands of years because of its curative effects and unique diagnosis and treatment system [3]. The combination of Zingiberis rhizoma (ZR) and Coptidis rhizoma (CR) is a classic TCM-based herb pair, which is recorded in “Treatise on Cold Pathogenic and Miscellaneous Diseases” (“Shang Han Za Bing Lun” in Chinese). This herb pair was mainly used for digestive system diseases according to ancient records but it is currently also used for its antitumor effects. However, the material basis and mechanisms of the antitumor actions of ZR-CR still need to be further explored.

A growing number of theories and practices show that network pharmacology can clarify the potential mechanisms of action of multicomponent and multitarget drugs by integrating multidisciplinary information and analyzing the effects via a network of actions at various levels [4]. Therefore, in our study, a network pharmacology approach was utilized to predict the antitumor mechanisms of ZR-CR. A surface plasmon resonance (SPR) assay was used to validate the predicted component-target affinity.

## 2. Materials and Methods

### 2.1. Collection of ZR and CR Information

Using TCMSP [5], TCMIP [6], and TCMID [7], and the keywords “Zingiberis rhizoma (Ganjiang in Chinese)” and “Coptidis rhizoma (Huanglian in Chinese),” compounds in ZR and CR were identified. The composition data of the compounds were obtained, and the datasets of the two Chinese medicine compounds were constructed after aggregation and removal of duplicates.

### 2.2. Collection of ZR and CR Targets

Similarity Ensemble Approach software (SEAware) [8, 9] was used to reverse target the chemical constituents of ZR and CR by setting the screening condition to human and then calculating and predicting the potential targets of ZR and CR. For targets of compounds that had not been collected by the SEAware, the compound name and SMILES code were queried using PubChem [10]; then, the targets of ingredients predicted using STITCH [11] and the Swiss Target Prediction database [12] were used to supplement the findings. All targets obtained were integrated, and the targets of all components were calibrated to the official name based on the Uniprot database [13].

### 2.3. Collection of Tumor Targets

GeneCards [14], NCBI-gene (https://www.ncbi.nlm.nih.gov/), and DisGeNET [15] databases were searched to identify tumor-related targets using keywords such as “tumor,” “cancer,” and “carcinoma.” The target names were unified using the Uniprot database [13] by integrating the calculated potential targets of ZR and CR with a set relevance score ≥ 1. This process enabled us to obtain the potential antitumor targets of the active components of ZR and CR. The R programming language was used to match the targets of the components with those of the disease and construct a Venn diagram.

### 2.4. Network Construction and Analysis

The target interactions of ZR and CR were introduced into the STRING database [16] to obtain protein–protein interactions, and the active ingredients and targets of ZR and CR were imported into the Cytoscape 3.6.1 software [17] to construct a comprehensive network diagram. The top 20 hub target networks were obtained using the Cytohubba module, and the NetworkAnalyzer module was used to analyze the degree and related parameters of the node. The potential transcription factors (TFs) based on the predicted antitumor targets of ZR and CR were investigated using the Metascape database (TRRUST module) [18, 19].

### 2.5. Pathway and Process Enrichment Analyses

Pathway and process enrichment analyses of the predicted antitumor targets of ZR and CR were conducted and visualized using the Metascape database [18]. The hub antitumor targets of ZR and CR were introduced into the DAVID database for GO analysis and KEGG pathway analysis [20, 21]. GO analysis was used to annotate and classify targets according to biological process (BP), molecular function (MF), and cellular component (CC). The top 20 terms were imported into the R programming language software for visual display.

### 2.6. Tumor Types Targeted by ZR and CR

The tumor-related targets of ZR and CR were imported into the Enrichr database [22] to identify the relationship between targets and diseases. The results were visualized using the R language software.

### 2.7. Molecular Docking

The hub target network was constructed by molecular docking between key targets and their corresponding components. The interaction between small molecules and targets was simulated using professional software to calculate their binding strengths as follows. The crystal structures of four hub targets (AKT1, EGFR, TP53, and VEGFA) were downloaded from the Protein Data Bank database [23] for molecular docking studies. The small molecules corresponding to the key target were generated in the lowest energy conformation. Then, the protein crystals and small molecules were introduced into the Molecular Operating Environment (MOE) software for structural preprocessing, and the dock module in the MOE and AutoDock Vina software was used to complete the molecular docking. The small molecule corresponding to the hub target albumin was oleic acid, which has a highly flexible long chain that might reduce the docking study accuracy, and therefore, we did not conduct a related experiment.

### 2.8. Materials and Buffer Preparation

Human AKT1, EGFR, and VEGF165 proteins were obtained from ACROBiosystems (Beijing, China), and human TP53 protein was purchased from Active Motif (Shanghai, China). Berberine (CAS: 2086-83-1), quercetin (CAS: 117-39-5), and daucosterol (CAS: 474-58-8) were purchased from Solarbio (Beijing, China), and ginkgetin (CAS: 481-46-9) and isoginkgetin (CAS: 548-19-6) were purchased from MedChemExpress (Shanghai, China). KH_2_PO_4_, NaCl, Na_2_HPO_4_, and KCl were obtained from Sinopharm Chemical Reagent (Shanghai, China), and dimethyl sulfoxide (DMSO) was purchased from Sangon Biotech (Shanghai, China).

The Series S Sensor Chip CM5 and Amine Coupling kit were obtained from GE Healthcare (Little Chalfont, UK). The prepared buffers were the running buffer (pH 7.4): 1 × PBS (2 mM KH_2_PO_4_, 10 mM Na_2_HPO_4_, 137 mM NaCl, and 2.7 mM KCl) with 0.05% Tween 20 and 5% DMSO and immobilization buffer (pH 4.0): 10 mM sodium acetate.

### 2.9. Affinity Test

The SPR assay was conducted using a Biacore 8K instrument (GE Healthcare) with the running buffer. Briefly, 50 *μ*g/mL of the target proteins in 10 mM NaAc (pH 4.5; Sinopharm Chemical Reagent, Shanghai, China) was then injected into the sample channel of the CM5 chips at a flow rate of 10 *μ*L/min to reach an immobilization level. The detection was performed according to the protocol provided by GE Healthcare. Gradient concentrations of the components (0, 1, 10, and 100 *μ*M) were dissolved in the running buffer and then injected into the channel at a flow rate of 30 *μ*L/min, with an association phase of 60 s, followed by a 90-second dissociation. The affinity was analyzed using the Biacore 8K software.

### 2.10. Hub Gene Expression and Survival Analysis in Patients

The relative gene expression level of 20 hub antitumor targets of ZR and CR was analyzed using the GEPIA database [24]. The differential expression between tumor and adjacent normal tissues for hub genes across all The Cancer Genome Atlas (TCGA) tumors was analyzed using the TIMER database [25]. The GEPIA database was also used to conduct overall survival and disease-free survival analyses based on gene expression. Tumor names as in TCGA were adopted in the analysis process (Table S1).

## 3. Results

### 3.1. Potential Tumor Targets of ZR and CR

**Figure 1:**
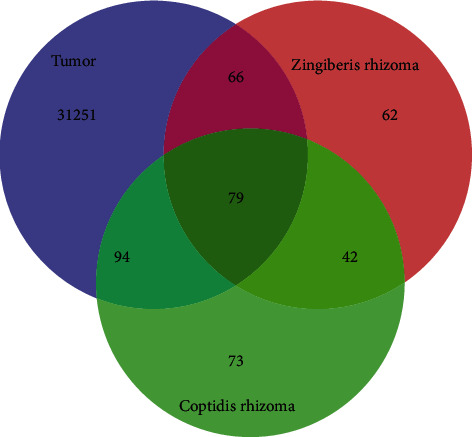
Intersection of Zingiberis rhizoma (ZR), Coptidis rhizoma (CR), and tumor targets was used to identify potential targets of ZR-CR against tumors.

We identified 157 and 55 chemical components of ZR and CR, respectively, for a total of 212 ingredients (Table S2). Then, 416 corresponding potential targets were selected, consisting of 249 and 288 targets in ZR and CR, respectively, which included 121 shared targets between ZR and CR. In addition, a total of 31490 tumor-related targets were identified. After intersecting the component and tumor targets, 239 common targets were obtained, including 79 targets that were shared by ZR and CR. The 239 common targets were considered potential targets for ZR and CR against tumors (Figure 1).

### 3.2. Protein–Protein Interaction and Component-Target Networks

**Figure 2:**
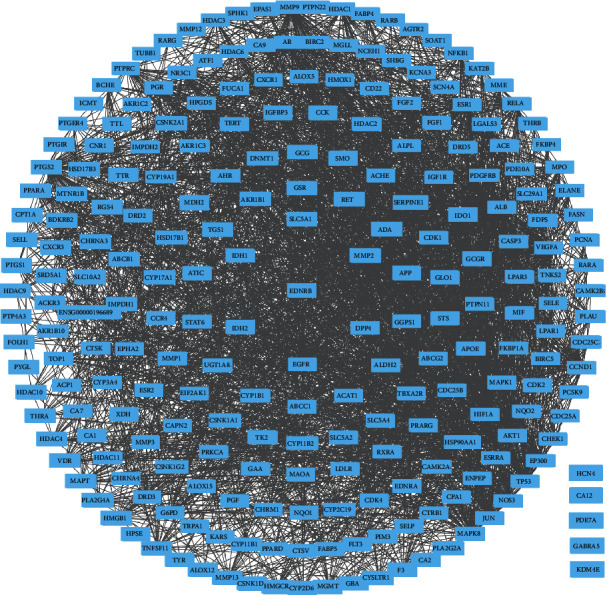
Protein–protein interaction network of potential targets of Zingiberis rhizoma (ZR) and Coptidis rhizoma (CR) against tumors.

**Figure 3:**
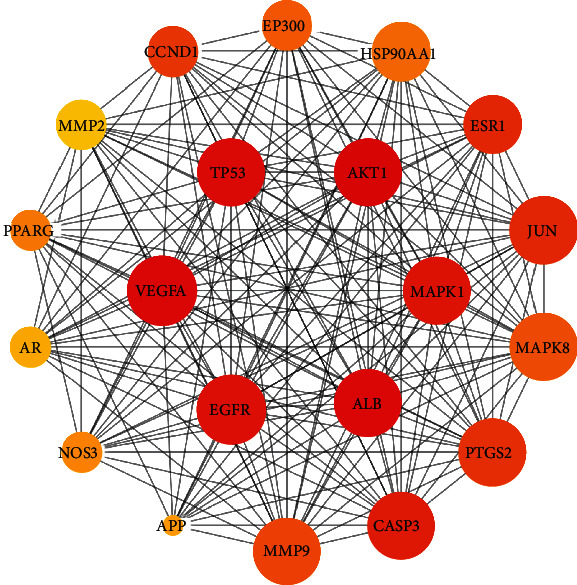
Protein–protein interaction network of top 20 hub targets of Zingiberis rhizoma (ZR) and Coptidis rhizoma (CR) against tumors.

**Figure 4:**
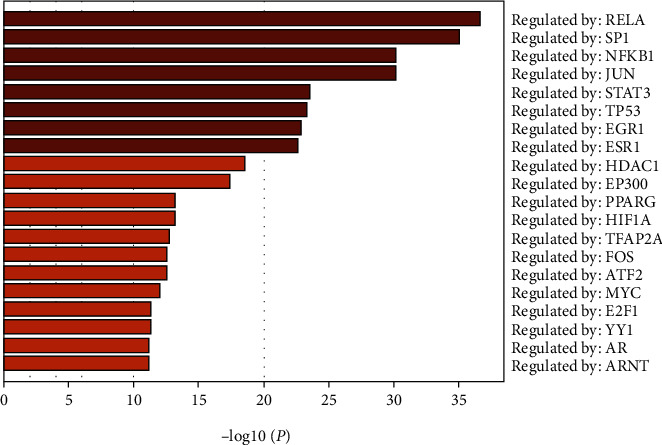
The 20 key transcription factors related to the antitumor targets of Zingiberis rhizoma (ZR) and Coptidis rhizoma (CR).

The protein–protein interaction network of potential targets of ZR and CR against tumors contained 239 nodes and 2814 edges (Figure 2). The hub target network shows the top 20 targets of ZR and CR against tumors (Figure 3 and Table 1). Nodes changed from orange to red and small to large, indicating that the degree gradually increased, and the importance of the targets was determined by the degree value. Upon analysis of 239 predicted antitumor targets of ZR and CR, 20 key potential TFs were identified (Figure 4).

**Figure 5:**
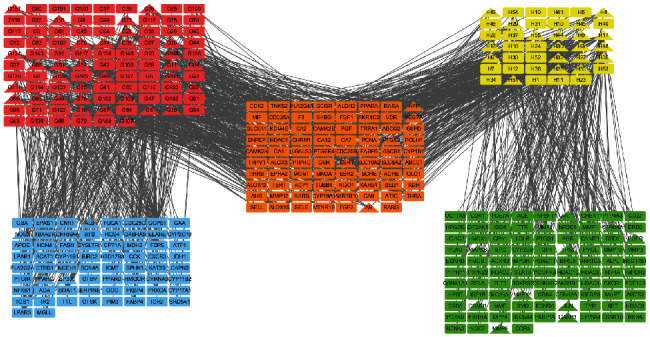
Network of Zingiberis rhizoma (ZR) and Coptidis rhizoma (CR) components corresponding to tumor targets. Red node, ZR component; yellow node, CR component; blue node, tumor target of ZR; green node, tumor target of CR; orange node, shared tumor target of ZR and CR; triangle node, hub component and target of ZR and CR.

**Figure 6:**
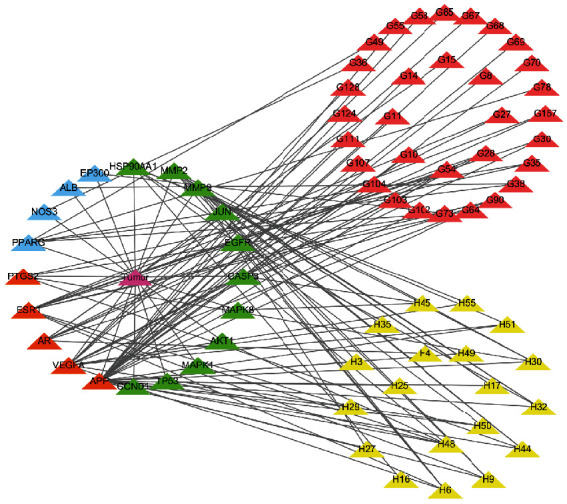
Hub network of Zingiberis rhizoma (ZR) and Coptidis rhizoma (CR) components corresponding to tumor targets. Red node, ZR component; yellow node, CR component; blue node, tumor target of ZR; green node, tumor target of CR; orange node, shared tumor target of ZR and CR.

The network of the components in ZR and CR corresponding to tumor targets contained 380 nodes and 1244 edges (Figure 5), and the network of hub components and targets contained 72 nodes and 117 edges (Figure 6), where the nodes represent the components of ZR and CR or the targets corresponding to the components, whereas the edges indicate interactions between components and targets.

### 3.3. Pathway and Process Enrichment

**Figure 7:**
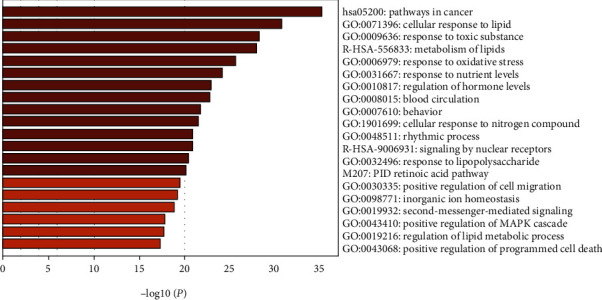
Top 20 terms in pathway and process enrichment analyses of the antitumor targets of Zingiberis rhizoma (ZR) and Coptidis rhizoma (CR).

**Figure 8:**
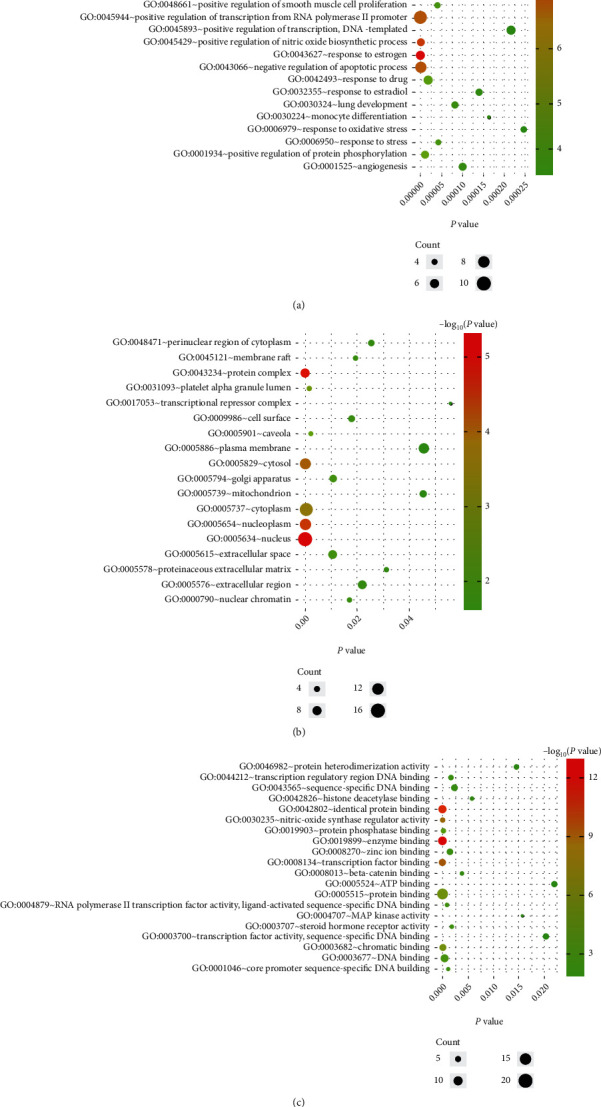
GO enrichment analysis of hub antitumor targets of Zingiberis rhizoma (ZR) and Coptidis rhizoma (CR). Top ranking (a) biological processes, (b) cellular components, and (c) molecular functions.

**Figure 9:**
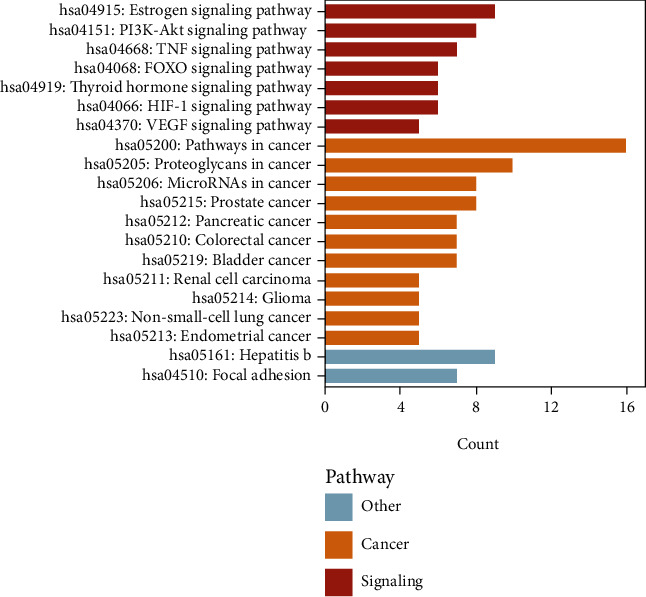
Top 20 terms in KEGG enrichment analyses of hub antitumor targets of Zingiberis rhizoma (ZR) and Coptidis rhizoma (CR).

**Figure 10:**
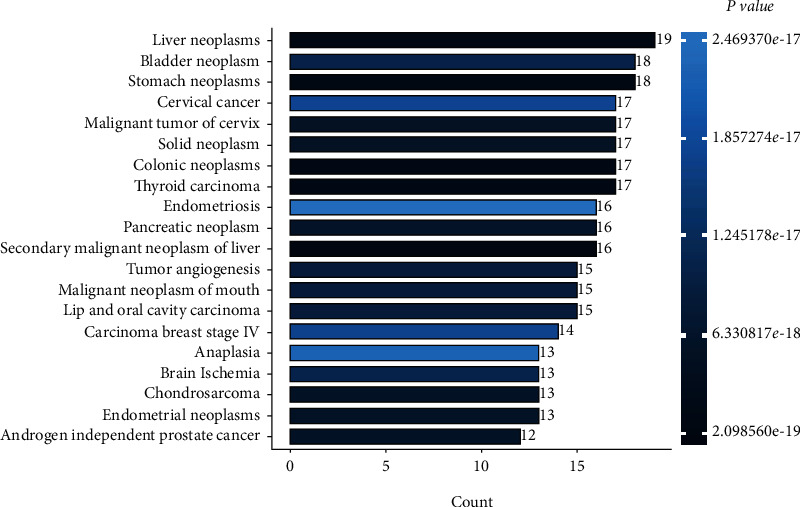
Top 20 disease types in Enrichr enrichment analysis of hub targets of Zingiberis rhizoma (ZR) and Coptidis rhizoma (CR; number behind bar indicates targets corresponding to disease).

The top 20 terms in the pathway and process enrichment analyses of the predicted antitumor targets of ZR and CR via the Metascape database are shown in Figure 7. The GO enrichment analysis indicated that the hub antitumor targets of ZR and CR are involved in 144, 18, and 42 BP-, CC-, and MF-related entries, respectively. The top-ranking entries based on *P* value are shown in Figure 8; the larger the dots, the more the number of enriched targets and the redder the dots, the smaller the *P* value. The top 20 KEGG enrichment entries of the hub antitumor targets of ZR and CR are shown in Figure 9. The top 20 disease types of Enrichr enrichment entries of the hub targets are shown in Figure 10, which contains multiple tumor types.

### 3.4. Molecular Docking between Components and Target Proteins

**Figure 11:**
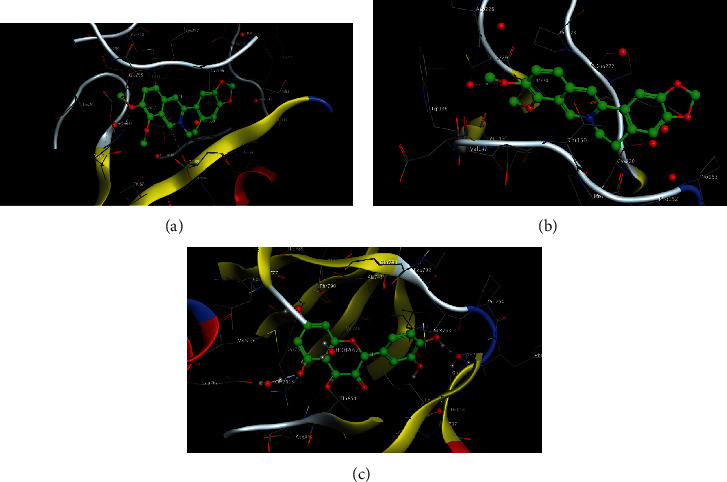
Partial view of molecular docking. (a) Berberine and AKT1 (binding free energy: −88.58 kCal/mol), (b) berberine and TP53 (binding free energy: −62.173 kCal/mol), and (c) quercetin and EGFR (binding free energy: −126.111 kCal/mol).

The MOE molecular docking scoring value was used to evaluate the interactions between the small molecules and proteins (Table 2); the smaller the scoring value, the stronger the interaction. The results showed that berberine and AKT1, berberine and TP53, and quercetin and EGFR may have stronger binding effects than observed with other interactions. The binding free energy of the first molecule docking posture of these three interactions is shown in Figure 11. Because numerous components corresponded to the VEGFA protein, AutoDock Vina was used for molecular docking. The top 10 terms identified by AutoDock Vina are shown in Table 3; the smaller the binding energy, the stronger the binding capacity of the target protein and component.

### 3.5. Affinity of Components for Target Proteins

**Figure 12:**
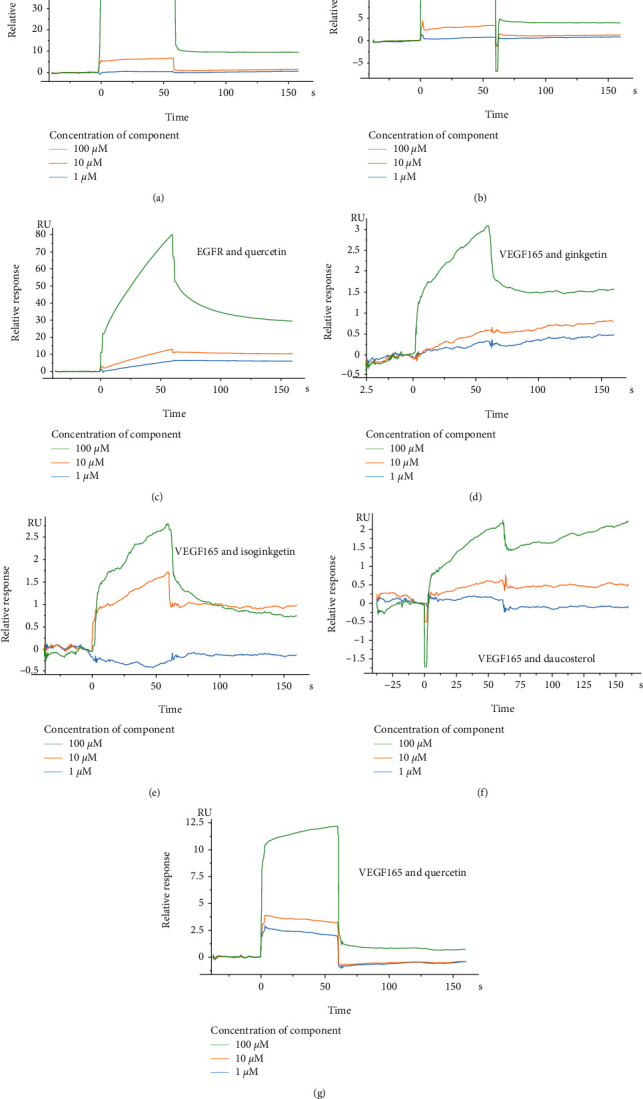
Relative response value of affinity test between (a) AKT1 and berberine, (b) TP53 and berberine, (c) EGFR and quercetin, (d) VEGF165 and ginkgetin, (e) VEGF165 and isoginkgetin, (f) VEGF165 and daucosterol, and (g) VEGF165 and quercetin by surface plasmon resonance assay.

The relative response values of the SPR affinity tests suggested that several components of ZR and CR can directly bind to its tumor target protein (Table 4 and Figure 12), and the affinity between ginkgetin, isoginkgetin, and daucosterol and their common target VEGF165 was weak.

### 3.6. Clinical Significance of Hub Targets

**Figure 13:**
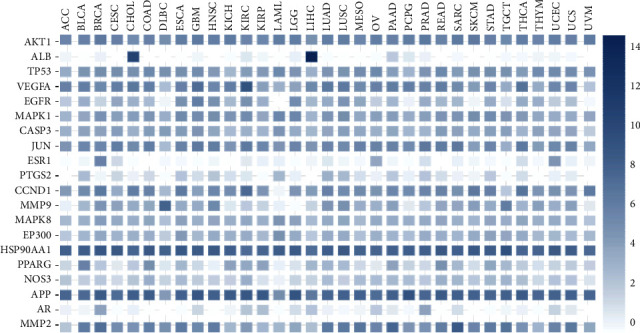
Relative expression level of 20 hub antitumor targets of Zingiberis rhizoma (ZR) and Coptidis rhizoma (CR) in multiple tumor tissues (numbers represent a comparison of relative gene expression).

**Figure 14:**
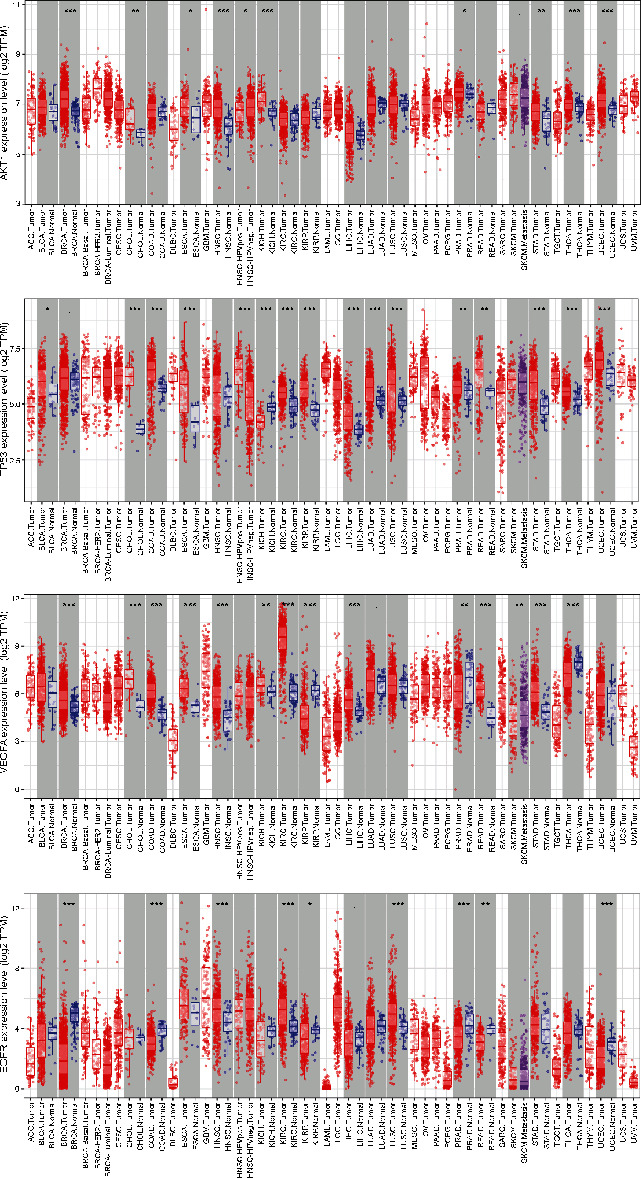
Differential gene expression of hub targets (AKT1, TP53, VEGFA, and EGFR) between tumor and adjacent normal tissues (*P* value: 0 ≤ ^∗∗∗^ < 0.001 ≤ ^∗∗^ < 0.01 ≤ ^∗^ < 0.05 ≤ .<0.1).

**Figure 15:**
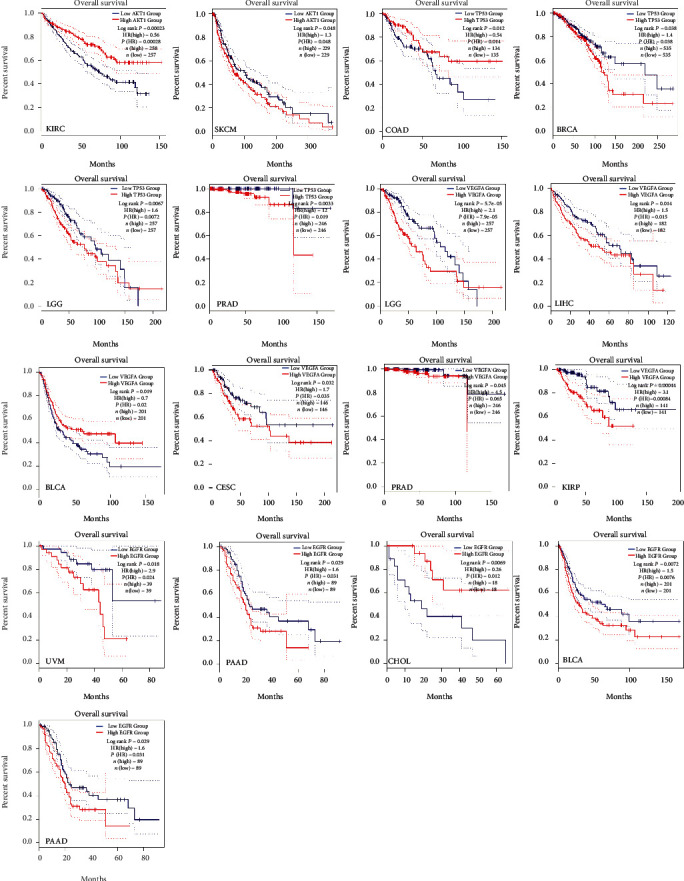
Prognostic value of hub targets (AKT1, TP53, VEGFA, and EGFR) in the overall survival of patients with tumors (HR: hazard ratios; statistical significance: log rank *P* value <0.05).

**Figure 16:**
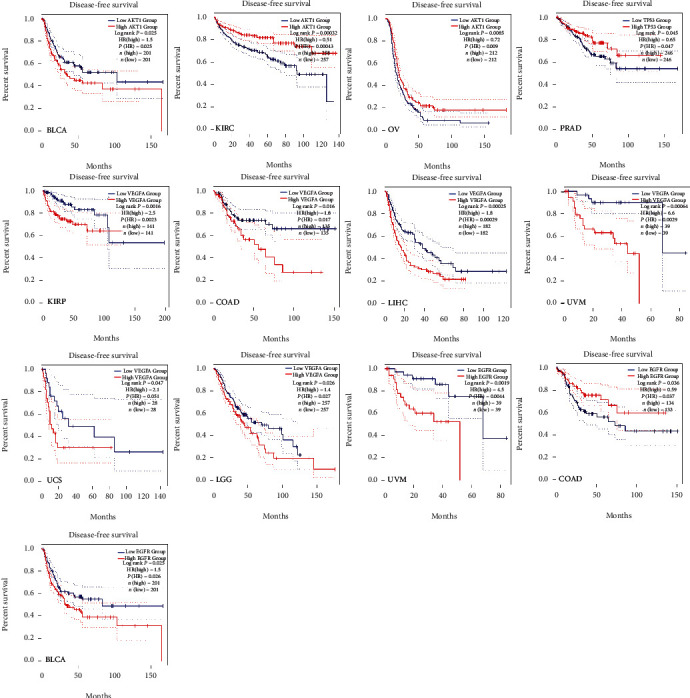
Prognostic value of hub targets (AKT1, TP53, VEGFA, and EGFR) in the disease-free survival of patients with tumors (HR: hazard ratios; statistical significance: log rank *P* value <0.05).

The relative gene expression levels of 20 hub antitumor targets of ZR and CR in different tumor tissues were compared. The profiles showed that different hub targets are differentially expressed in multiple tumor tissues (Figure 13), and *AKT1*, *TP53*, *VEGFA*, and *EGFR* are differentially expressed between multiple tumors and adjacent normal tissues (Figure 14), such as breast invasive carcinoma, cholangiocarcinoma, prostate adenocarcinoma, and thyroid carcinoma. The results also showed that hub gene expression levels are associated with unfavorable overall survival and disease-free survival in patients with multiple tumor types (Figures 15 and 16), such as patients with kidney renal clear cell carcinoma, pancreatic adenocarcinoma, and brain lower grade glioma.

## 4. Discussion

Presently, the ZR-CR herbal pair is widely used in the field of antitumor TCM. In this study, the mechanisms of antitumor action of the ZR-CR herbal pair were investigated using a network pharmacology approach. The network pharmacology analysis suggested that the active components of ZR and CR, such as gingerdione, shogaol, zingerone, sitosterol, daucosterol, ginkgetin, isoginkgetin, berberine, quercetin, noroxyhydrastinine, chlorogenic acid, vanillic acid, and ethyl caffeate, identified using the TCMSP, TCMIP, and TCMID platforms, could serve as effective therapeutic agents for the treatment of tumors via multiple mechanisms.

The component-target network showed that, although multiple components corresponded to a single target, a single component also corresponded to multiple targets, indicating that the effects of the ZR-CR herbal pair on tumors are mediated through multiple components and multiple targets. Among the numerous targets, hub targets such as AKT1, TP53, VEGFA, EGFR, and MAPK1 may play important roles. Furthermore, hub targets such as TP53, JUN, ESR1, EP300, PPARG, and AR are also TFs that may be involved in the regulation of their target genes.

Pathway and process enrichment analyses suggested that the ZR-CR herbal pair is involved in mechanisms such as the metabolism of lipids, response to oxidative stress, and regulation of hormone levels, cell migration, MAPK cascade, and programmed cell death to exert antitumor effects. The enrichment analyses of hub targets suggested that ZR and CR are involved in estrogen, MAPK, PI3K-AKT, TNF, FOXO, HIF-1, and VEGF signaling pathways, which are also closely related to antitumor activities. For example, abnormalities in MAPK signaling play a crucial part in the progression of cancer [26]. FOXO is a subfamily of the forkhead transcription factor family that is involved in cell fate decisions [27]. HIF-1 functions as a signaling hub that coordinates the activities of many TFs and signaling molecules that affect tumorigenesis [28].

Disease type enrichment analysis showed that the ZR-CR herbal pair plays a role in tumors of the digestive, urinary, and gynecological systems. This finding is also consistent with the extensive application of ZR and CR in the treatment of digestive system diseases and tumors in TCM [29, 30].

Molecular docking analysis and SPR assays were conducted based on the hub targets of ZR and CR against tumors to preliminarily screen and validate our predictions. The results suggested that berberine directly binds to AKT1 and TP53 and quercetin to EGFR and VEGF165. Ginkgetin, isoginkgetin, and daucosterol directly bind to VEGF165 but with weak affinities. Although affinity is a crucial functional factor of pharmacological activity, its strength does not necessarily determine the potency of the effect. For example, one study investigated the effect of affinity and antigen internalization on the uptake and penetration of anti-HER2 antibodies in solid tumors. The findings suggested that antibodies with lower affinity penetrate tumors more effectively when rates of antibody-antigen dissociation are higher than those of antigen internalization [31]. Some studies have shown that these identified compounds also indirectly affect target proteins and exhibit antitumor activities. For example, berberine induces p53 expression and thereby decreases the mitochondrial membrane potential and induces cytochrome C release [32]. The AKT-related mitochondrial pathway may be partly involved in berberine-induced apoptosis of gastric cancer cells [33]. Quercetin prevents prostate cancer progression in an *in vivo* model by inhibiting EGFR signaling [34]. In addition, the combination of quercetin and metformin exerts synergistic antitumor effects by inhibiting the VEGF/PI3K/AKT pathway [35]. Ginkgetin suppresses VEGF-mediated angiogenesis during cancer development [36]. Furthermore, isoginkgetin, an isomer of ginkgetin, markedly decreases MMP9 expression and invasion by inhibiting PI3K/AKT [37]. Daucosterol linoleate suppresses VEGF, MMP2, and MMP9 expression in breast cancer [38].

These previous studies mostly focused on evaluating pharmacological parameters, functional phenotype changes, and molecular mechanisms limited to some classical signaling pathways. Consequently, direct interactions between active molecules and target proteins are often not considered [39]. The current results show the direct binding of small-molecule compounds to tumor-related proteins, which confirmed that ZR and CR components act on multiple tumors.

Furthermore, hub targets, such as AKT1, TP53, VEGFA, and EGFR, were shown to be differentially expressed between multiple tumors and adjacent normal tissues, and their expression levels can impact overall survival and disease-free survival in patients with tumors. These pan-cancer analyses are expected to benefit the clinical application of the ZR-CR herbal pair. The hub targets identified in our study are considered targets of currently available antitumor drugs such as EGFR inhibitors (gefitinib, erlotinib, and lapatinib) [40]. Several anti-VEGF drugs have also been approved for certain advanced cancers, but they have only exhibited limited benefits on the overall survival of patients with tumors and have rarely resulted in durable responses [41]. TCM components often exhibit less potent effects than modern drugs with clear targets, but the overall efficacy advantage of TCM preparations for tumor treatment is not only based on a simple additive effect of multiple “weak effects.” In vitro and in vivo EGFR mutant non-small-cell lung cancer models have shown that 20% of individual effective drug doses can completely block MAPK signaling when used in RAF+MEK+ERK or EGFR+RAF+MEK+ERK inhibitor combinations [42]. Our findings suggest that the synergistic antitumor effect of the compounds in ZR and CR deserves more detailed exploration.

Although some components of ZR-CR with activity against tumors in the hub network were not analyzed further, they are not unimportant. Related studies have been reported, such as one that showed that shogaol causes cancer cell death by inducing cell cycle arrest and apoptosis [43]. Additionally, the antitumor activity of chlorogenic acid is mediated through the induction of cancer cell differentiation [44], and vanillic acid inhibits hypoxia-induced HIF-1*α* expression in various human cancer cell lines [45]. Therefore, these components of the ZR-CR herbal pair also warrant further experimental and clinical studies.

## 5. Conclusions

The active ingredients of the ZR-CR herbal pair and their underlying antitumor mechanisms were partly identified. The network pharmacology approach revealed that the components of ZR-CR, such as shogaol, daucosterol, ginkgetin, berberine, quercetin, chlorogenic acid, and vanillic acid, have potential efficacy as antitumor agents; they mediate their activity via multiple mechanisms, such as MAPK, PI3K-AKT, TNF, FOXO, HIF-1, and VEGF signaling pathways. The ZR-CR herbal pair could be useful in the treatment of tumors, such as those of the digestive, urinary, and gynecological systems. This network-based investigation could facilitate the elucidation of the mechanisms of action of the ZR-CR herbal pair against tumors. The molecular docking approach and SPR assay identified some components of ZR-CR that directly bind to hub target proteins to exert their antitumor effects. The gene expression levels of hub targets of the ZR-CR herbal pair are associated with overall survival and disease-free survival in patients with multiple tumor types. Finally, the antitumor components of the ZR-CR warrant further experimental and clinical investigations, as they have not been discussed adequately in our study.

## Figures and Tables

**Table 1:** Top 20 hub targets of Zingiberis rhizoma (ZR) and Coptidis rhizoma (CR) against tumors obtained from the protein–protein interaction network.

Rank	Target name	Degree value
1	AKT1	135
2	ALB	110
3	TP53	108
4	VEGFA	106
5	EGFR	96
6	MAPK1	90
7	CASP3	86
8	JUN	83
9	ESR1	82
10	PTGS2	81
11	CCND1	73
12	MMP9	72
13	MAPK8	71
14	EP300	70
15	HSP90AA1	68
16	PPARG	66
17	NOS3	62
18	APP	57
19	AR	56
20	MMP2	54

**Table 2:** Molecular Operating Environment (MOE) molecular docking results.

Component name	Target protein	Scoring value
Berberine	AKT1	-6.86326
		-6.83849
		-6.79205

Berberine	TP53	-6.79754
		-6.75291
		-6.52506

Quercetin	EGFR	-6.79373
		-6.73849
		-6.72856

Ethyl caffeate	EGFR	-5.75152
		-5.74198
		-5.73384

**Table 3:** Top 10 molecular docking terms identified by AutoDock Vina.

Rank	Component name	Target protein	Binding energy
1	Ginkgetin	VEGFA	-7.1
2	Isoginkgetin		-7.0
3	6-O-E-Feruloylajugol		-6.2
4	Daucosterol		-5.6
5	5-(beta-D-Glucopyranosyloxy)-2-hydroxybenzoic acid		-5.5
6	(2R,3R,4R,5S,6R)-2-[2-(3,4-Dihydroxyphenyl)ethoxy]-6-(hydroxymethyl)oxane-3,4,5-triol		-5.5
7	Quercetin		-5.5
8	Sexangularetin		-5.4
9	[(2S)-2-hydroxy-3-[(2R,3R,4S,5R,6R)-3,4,5-trihydroxy-6-[[(2S,3R,4S,5R,6R)-3,4,5-trihydroxy-6-(hydroxymethyl)tetrahydropyran-2-yl]oxymethyl]tetrahydropyran-2-yl]oxy-propyl](9Z,12Z)-octadeca-9,12-dienoate		-5.2
10	Dopaol beta-D-glucoside		-5.1

**Table 4:** Affinity test between component and tumor target using surface plasmon resonance (SPR) assay.

Ligand name	Analyte name	Analyte concentration	Relative response (RU)
AKT1	Berberine	1 *μ*M	-1.7
		10 *μ*M	4.6
		100 *μ*M	63.8

TP53	Berberine	1 *μ*M	0.8
		10 *μ*M	3.4
		100 *μ*M	22.7

EGFR	Quercetin	1 *μ*M	5.8
		10 *μ*M	12.4
		100 *μ*M	77.0

VEGF165	Ginkgetin	1 *μ*M	1.0
		10 *μ*M	1.3
		100 *μ*M	3.7

VEGF165	Isoginkgetin	1 *μ*M	0.3
		10 *μ*M	2.2
		100 *μ*M	3.3

VEGF165	Daucosterol	1 *μ*M	0.0
		10 *μ*M	0.5
		100 *μ*M	2.0

VEGF165	Quercetin	1 *μ*M	2.5
		10 *μ*M	3.5
		100 *μ*M	12.1

## Data Availability

The datasets used and/or analyzed during the current study are available from the corresponding author on reasonable request.

## Supplementary Materials

Supplementary Materials Table S1: tumor name in TCGA. Table S2: components of Zingiberis rhizoma and Coptidis rhizome.
