# Supplementary material for: Exploring the Antitumor Mechanisms of Zingiberis Rhizoma Combined with Coptidis Rhizoma Using a Network Pharmacology Approach
Source: Biomed Res Int. 2020 Dec 24;2020:8887982. doi: 10.1155/2020/8887982 (PMC7781700; doi:10.1155/2020/8887982)
Supplement: Supplementary Materials — Table S1: tumor name in TCGA. Table S2: components of Zingiberis rhizoma and Coptidis rhizome. [file 8887982.f1.docx]

**Supplementary Material**

Table S1. Tumor name in TCGA.

| **TCGA** | **Detail** |
| --- | --- |
| **ACC** | Adrenocortical carcinoma |
| **BLCA** | Bladder urothelial carcinoma |
| **BRCA** | Breast invasive carcinoma |
| **CESC** | Cervical squamous cell carcinoma and endocervical adenocarcinoma |
| **CHOL** | Cholangio carcinoma |
| **COAD** | Colon adenocarcinoma |
| **DLBC** | Lymphoid neoplasm diffuse large B-cell lymphoma |
| **ESCA** | Esophageal carcinoma |
| **GBM** | Glioblastoma multiforme |
| **HNSC** | Head and neck squamous cell carcinoma |
| **KICH** | Kidney chromophobe |
| **KIRC** | Kidney renal clear cell carcinoma |
| **KIRP** | Kidney renal papillary cell carcinoma |
| **LAML** | Acute myeloid leukemia |
| **LGG** | Brain lower grade glioma |
| **LIHC** | Liver hepatocellular carcinoma |
| **LUAD** | Lung adenocarcinoma |
| **LUSC** | Lung squamous cell carcinoma |
| **MESO** | Mesothelioma |
| **OV** | Ovarian serous cystadenocarcinoma |
| **PAAD** | Pancreatic adenocarcinoma |
| **PCPG** | Pheochromocytoma and paraganglioma |
| **PRAD** | Prostate adenocarcinoma |
| **READ** | Rectum adenocarcinoma |
| **SARC** | Sarcoma |
| **SKCM** | Skin cutaneous melanoma |
| **STAD** | Stomach adenocarcinoma |
| **TGCT** | Testicular germ cell tumors |
| **THCA** | Thyroid carcinoma |
| **THYM** | Thymoma |
| **UCEC** | Uterine corpus endometrial carcinoma |
| **UCS** | Uterine carcinosarcoma |
| **UVM** | Uveal melanoma |

Table S2. Components of Zingiberis rhizoma and Coptidis rhizome.

| **SMILES** | **ID** | **Chemical compound** | **Number** |
| --- | --- | --- | --- |
| **Zingiberis Rhizoma (ganjiang)** |  |  |  |
| O[C@@H](CCCCCCC)C | 11286536 | (R)-Nonan-2-ol | G1 |
| [C@H]1(C(C)C)CC=C(C=C1)C | 442482 | (R)-(-)-alpha-Phellandrene | G2 |
| O[C@@H](CCCCCCCCC)CC(=O)CCc1cc(OC)c(O)cc1 | 168115 | (10)-Gingerol | G3 |
| [C@H]1([C@H](CCC=C(C)C)C)CCC(=C)C=C1 | 12315492 | (-)-beta-Sesquiphellandrene | G4 |
| C(c1ccc(cc1)C)(C)C | 7463 | P-Cymene | G5 |
| O=CCCCCCCCC | 31289 | Nonanal | G6 |
| S1SCC(N(C)C)C1 | 15402 | Nereistoxin | G7 |
| O=C(CCCCC)CC(=O)CCc1cc(OC)c(O)cc1 | 162952 | Gingerdione | G8 |
| O1[C@@]2([C@@H]([C@@]3([C@H](C(CCC3)(C)C)CC2)C)C/C=C(\CC=O)/C=O)C1 | 21668974 | Aframodial | G9 |
| O([C@@H](C[C@H](OC(=O)C)CCc1cc(OC)c(O)cc1)CCc1cc(OC)c(O)c(OC)c1)C(=O)C | 5316611 | 1-(3,5-Dimethoxy-4-hydroxyphenyl)-7-(3-methoxy-4-hydroxyphenyl)-3,5-diacetoxyheptane | G10 |
| O([C@@H](C[C@@H](OC(=O)C)CCc1cc(OC)c(O)cc1)CCc1cc(OC)c(O)cc1)C(=O)C | 5319377 | [(3R,5S)-5-Acetyloxy-1,7-bis(4-hydroxy-3-methoxyphenyl)heptan-3-yl] acetate | G11 |
| O1[C@]2(CC[C@@H](C1)C)O[C@@H]1[C@@H]([C@@H]2C)[C@@]2([C@@H](C1)[C@H]1[C@H](CC2)[C@@]2(C(=CC1)C[C@H](CC2)O[C@@H]1O[C@@H]([C@H]([C@@H]([C@H]1O[C@@H]1O[C@H]([C@@H]([C@H]([C@H]1O)O)O)C)O)O[C@@H]1O[C@@H]([C@H]([C@@H]([C@H]1O)O)O)CO)CO)C)C | MOL002517 | Zingiberoside A3 | G12 |
| C1=C(C=C[C@H](C1)[C@@H](C)CCC=C(C)C)C | MOL000895 | Zingiberene | G13 |
| c1(cc(c(cc1)O)OC)CCC(=O)C | MOL002516 | zingerone | G14 |
| [C@@H]12[C@@H]([C@@]3([C@@H](CC2)CC(=O)C=C3)C)CC[C@]2([C@H]1CC[C@]2(C)O)C | MOL002473 | ZINC04081584 | G15 |
| [C@@H](CCC[C@@H](CCCC(=O)C)C)(CCCC(C)C)C | MOL001899 | ZINC02169908 | G16 |
| [C@@](CC/C=C(/CCC=C(C)C)\C)(C=C)(C)O | MOL000119 | ZINC02040970 | G17 |
| [C@@]12([C@@H](C([C@H](C1)CC2)(C)C)O)C | MOL002458 | ZINC01850974 | G18 |
| c1(cc(c(cc1)C)O)[C@@H](CCC=C(C)C)C | MOL002513 | Xanthorrhizol | G19 |
| C(CCCCCCCCC)CCCCCCCCC | MOL001619 | UPL | G20 |
| C[C@@]12[C@H]3[C@@H]1C[C@H](C3)C2(C)C | MOL000913 | Tricyclene | G21 |
| CCCCCCCCCCCCCC | MOL000886 | Tetradecane | G22 |
| c1(ccc(cc1)OC)CC=C | MOL002361 | Terragon | G23 |
| C1(=CC=C(CC1)C)C(C)C | MOL000911 | Terpilene | G24 |
| C1(=C(C)C)CCC(=CC1)C | MOL000264 | Tereben | G25 |
| C(=CCCC(=O)C)(C)C | MOL002496 | Sulcatone | G26 |
| [C@H]1(CC[C@]2(C(=CC[C@H]3[C@@H]4CC[C@H]([C@@H](CC[C@H](C(C)C)CC)C)[C@@]4(C)CC[C@H]23)C1)C)O | MOL000359 | Sitosterol | G27 |
| [C@H]12[C@H]3[C@@H]([C@@]4(C(=CC3)C[C@H](CC4)O[C@@H]3O[C@@H]([C@H]([C@@H]([C@H]3O)O)O)CO)C)CC[C@@]1([C@H](CC2)[C@@H](CC[C@H](C(C)C)CC)C)C | MOL000357 | Sitogluside | G28 |
| [C@]1([C@@H](C(=O)[C@@H](CC1)C(C)C)C(=C)C)(C=C)C | MOL002515 | Shyobunone | G29 |
| c12c(c(c(cc1O)O)OC)oc(c(c2=O)O)c1ccc(cc1)O | MOL002514 | Sexangularetin | G30 |
| C(=C\CC=C(C)C)(/C=C)\C | MOL000201 | p-Ocimene | G31 |
| CC(C)(O)[C@H]1C=CC(=CC1)C | MOL002474 | p-Mentha-1,5-dien-8-ol | G32 |
| C([C@@H](C)O)O | MOL002498 | PGR | G33 |
| [C@@](C)(C=C)(CC/C=C(/C)\CCC=C(C)C)O | MOL002504 | Peruviol | G34 |
| C(=O)(CCCCCCCCCCCCCC)O | MOL001396 | Pentadecylic acid | G35 |
| C(=O)(CCCCCCCCCCCCCCC)O | MOL000069 | Palmitic acid | G36 |
| C(CCCC)CCC=O | MOL000710 | OYA | G37 |
| C(C/C=C\CCCCCCCC)CCCCCC(=O)O | MOL000675 | Oleic acid | G38 |
| CCCCCCCCCCCCCCCCCC | MOL001394 | Oktadekan | G39 |
| c1(c(cccc1)C)C(C)C | MOL000712 | o-Cymol | G40 |
| C(=C\COC(=O)C)(/CCC=C(C)C)\C | MOL000128 | Nerylacetate | G41 |
| C(=C\COC(=O)CC)(\CCC=C(C)C)/C | MOL002472 | Neryl propionate | G42 |
| C(=C\COC(=O)C)(\CCC=C(C)C)/C | MOL000597 | Neryl acetate | G43 |
| C(=C\C=O)(\CCC=C(C)C)/C | MOL000127 | Neral | G44 |
| C(=C)(CCC=C(C)C)C=C | MOL000197 | Myrcene | G45 |
| C1(=CCC(=CC1)C)C(C)C | MOL000202 | Moslene | G46 |
| CC(=O)CCCCCCCCC | MOL000924 | Mnk | G47 |
| C(C=C)(C)(C)O | MOL002480 | Methylbutenol | G48 |
| C(=O)(CCCCCCCCCCCCCCC)OC | MOL000879 | Methyl palmitate | G49 |
| [C@@H]1(CNC[C@H]([C@H]1O)O)O | MOL002512 | Mesotrihydroxypiperidine | G50 |
| CCCCCCCCCCCCCCCCCCCC | MOL000868 | LFA | G51 |
| C(=O)(C)O[C@@H]1C[C@@H]2CC[C@]1(C2(C)C)C | MOL000196 | L-Bornyl acetate | G52 |
| C(C(C)C)C=O | MOL002484 | Isovaleral | G53 |
| c1(cc(c2c(c1)oc(cc2=O)c1cc(c(cc1)OC)c1c2oc(cc(=O)c2c(cc1O)O)c1ccc(cc1)OC)O)O | MOL002511 | Isoginkgetin | G54 |
| C(CC(=O)/C=C/CCc1cc(c(cc1)O)OC)c1cc(c(c(c1)OC)O)OC | MOL002470 | Isogingerenone-B | G55 |
| [C@H]12C(=C(CCCC1(C)C)C)CCC(=C2)C | MOL002475 | Himachalene | G56 |
| C(CCC)CC=O | MOL000666 | Hexanal | G57 |
| O[C@@H](CCc1cc(OC)c(O)cc1)CC(=O)CCc1cc(OC)c(O)cc1 | MOL002468 | Hexahydrocurcumin | G58 |
| O=Cc1c(O)c(c2c(c1)c1c([nH]2)cccc1)CC=C(C)C | MOL002510 | Heptaphylline | G59 |
| CCCCCCCCCCCCCCCCC | MOL000867 | Heptadekan | G60 |
| CCCCCCCCCCCCCCCCCCCCC | MOL000869 | Henicosane | G61 |
| C1(=CC[C@@H](CC1)C(=C)C)C | MOL000023 | Hemo-sol | G62 |
| C12=C([C@H](CCC(=C(C)C)C1)C)CC[C@H]2C | MOL000170 | Guaiene | G63 |
| c1(cc(c2c(c1)oc(cc2=O)c1cc(c(cc1)OC)c1c(cc(c2c1oc(cc2=O)c1ccc(cc1)O)O)O)O)OC | MOL002509 | Ginkgetin | G64 |
| O1[C@@H]([C@H](O)[C@](OC(=O)CCCCCCC/C=C\CCCCCCCC)(O)C(O)(O)[C@@H]1OCCC)CO | MOL002466 | Gingerglycolipid C_qt | G65 |
| O1[C@@H]([C@H](O)[C@](OC(=O)CCCCCCC/C=C\C/C=C\C/C=C\CC)(O)C(O)(O)[C@@H]1OCCC)CO | MOL002462 | Gingerglycolipid A_qt | G66 |
| O1[C@@H]([C@H](O)[C@](OC(=O)CCCCCCC/C=C\C/C=C\C/C=C\CC)(O)C(O)(O)[C@@H]1OCCC)CO[C@H]1O[C@@H]([C@H](O)[C@H](O)[C@@H]1O)CO | MOL002461 | Gingerglycolipid A | G67 |
| c1(c(ccc(c1)CCC(=O)/C=C/CCc1ccc(cc1)O)O)OC | MOL002508 | Gingerenone-C | G68 |
| c1(c(ccc(c1)CCC(=O)/C=C/CCc1cc(c(c(c1)OC)O)OC)O)OC | MOL002507 | Gingerenone B | G69 |
| C(c1cc(c(cc1)O)OC)CC(=O)/C=C/CCc1cc(c(cc1)O)OC | MOL002506 | Gingerenone A | G70 |
| C(=C\CO)(/CCC=C(C)C)\C | MOL000123 | Geraniol | G71 |
| C=CC(=C)CC/C=C(/CCC=C(C)C)\C | MOL000479 | Farnesene | G72 |
| C(C/C=C\C/C=C\CCCCC)CCCCCC(=O)O | MOL000131 | EIC | G73 |
| CCCCCCCCCC | MOL000863 | Dekan | G74 |
| C(CCCCC)CCCC=O | MOL000121 | Decanal | G75 |
| C(CCCCCO)CCCCO | MOL002519 | Decamethylenediol | G76 |
| [C@@H]12C(C(=C)[C@@H](CC1)C2)(C)C | MOL000019 | D-Camphene | G77 |
| c1(c(C(=O)OCCCC)cccc1)C(=O)OCCCC | MOL000676 | DBP | G78 |
| [C@H]1(CC2=CC[C@@]3([C@@H]([C@]2(CC1)C)C[C@H]([C@]1([C@]3(O)CC[C@@H]1O)CC(=O)C)OC(=O)/C=C(/C(C)C)\C)O)O[C@@H]1O[C@@H]([C@@H]([C@@H](C1)OC)O[C@@H]1O[C@@H]([C@@H]([C@@H](C1)OC)O[C@@H]1O[C@@H]([C@@H]([C@@H](C1)OC)O[C@@H]1O[C@@H]([C@H]([C@@H]([C@H]1O)O)O)CO)C)C)C | MOL002503 | Cynanuriculoside A | G79 |
| C(=O)(C1CC1)C1CC1 | MOL002457 | Cyclopropyl ketone | G80 |
| [C@]12([C@@H]3[C@@H]([C@H]1CC=C3C)[C@@H](CC2)C(C)C)C | MOL001600 | Copaene | G81 |
| [C@H]12[C@@H]3[C@H]([C@]1(CC[C@H]3C(C)C)C)CC=C2C | MOL002502 | Copaene (1) | G82 |
| C/C(=C\C=C\C(=C\C=C\C=C(/C)\C=C\C=C(/C)\C=O)\C)/C=C/C1=C(C)C[C@@H](CC1(C)C)O | MOL000765 | Citraurin beta | G83 |
| C(=O)/C=C(/CCC=C(C)C)\C | MOL000124 | Citral | G84 |
| C=CC(=C)CC/C=C(\CCC=C(C)C)/C | MOL000926 | cis-beta-Farnesene | G85 |
| [C@H]1([C@@](CC[C@H](C1)C(=C)C)(C=C)C)C(=C)C | MOL000204 | -cis-.beta.-Elemene diastereomer | G86 |
| C1C[C@]2([C@@H](C[C@@H]1[C@@]2(CCC=C(C)C)C)O)C | MOL002500 | Campherenol | G87 |
| c1(c(c2c(cc1O)C(=O)OC[C@H]1O[C@H](OC(=O)c3cc(c(c(c3)O)O)O)[C@H]3[C@H]([C@@H]1OC(=O)C1=CC(=O)[C@]4(C([C@@H]1c1c(C(=O)O3)cc(c(c1O4)O)O)(O)O)O)OC(=O)c1c2c(c(c(c1)O)O)O)O)O | MOL002505 | C10230 | G88 |
| [C@]12(C=C[C@@H]([C@@H]1C2)C)C(C)C | MOL002499 | Bicyclo[3. 1. 0]hex-2-ene,4-methyl-1-(1-methylethyl)- | G89 |
| [C@H]12[C@H]3[C@@H]([C@@]4(C(=CC3)C[C@H](CC4)O)C)CC[C@@]1([C@H](CC2)[C@@H](CC[C@H](C(C)C)CC)C)C | MOL000358 | beta-Sitosterol | G90 |
| [C@H](CCC=C(C)C)(CCO)C | MOL002522 | beta-Rhodinol | G91 |
| C1[C@@H](CC[C@]2(CCCC(=C)[C@H]12)C)C(C)(C)O | MOL000032 | beta-Eudesmol | G92 |
| C1=C(CC=C(C1)[C@@H](CCCC(=C)C)C)C | MOL002521 | beta-Curcumene | G93 |
| [C@@H](CCC=C(C)C)(CCO)C | MOL000267 | beta-Citronellol | G94 |
| C=C(CCC=C(C)C)[C@H]1CC=C(CC1)C | MOL000968 | beta-Bisabolene | G95 |
| C=C/C(=C/C/C=C(/CCC=C(C)C)\C)/C | MOL000932 | alpha-Farnesene | G96 |
| c1(ccc(cc1)C)[C@@H](CCC=C(C)C)C | MOL000027 | alpha-Curcumene | G97 |
| [C@@]123[C@@H]([C@H]1C(=CC3)C)[C@@H](CC[C@H]2C)C(C)C | MOL002085 | alpha-Cubebene | G98 |
| [C@H]12C[C@H](CC=C1CO)C2(C)C | MOL001121 | 19894-97-4 | G99 |
| C(c1cc(c(cc1)O)OC)CC(=O)C[C@H](O)CCCCCCCCCCC | MOL002469 | 12-Gingerol | G100 |
| C1C[C@@]([C@]2([C@]([C@@H]1O)(CC[C@H]([C@@H]2O)[C@](O)(COC(=O)C)OC(=O)C)C)C)(C)O | MOL002460 | 12,13-Di-acetoxyl-1,4,6,11-eudesmanetetol | G101 |
| C(c1cc(c(cc1)O)OC)CC(=O)/C=C(\O)/CCCCCCCCC | MOL002454 | 10-Gingerdione | G102 |
| O1[C@@H]([C@H](O)[C@](OC(=O)CCCCCCC/C=C\CCCCCCCC)(O)C(O)(O)[C@@H]1OCCC)CO[C@H]1O[C@@H]([C@H](O)[C@H](O)[C@@H]1O)CO | MOL002465 | 9-Octadecenoic acid (Z)-, 3-((6-O-alpha-D-galactopyranosyl-beta-D-galactopyranosyl)oxy)-2-hydroxypropyl ester, (S)- | G103 |
| c1(cc(c(cc1)O)OC)CCC(=O)/C=C/CCCCC | MOL002495 | 6-Shogaol | G104 |
| S(=O)(=O)(O)[C@@H](CCCCC)CC(=O)CCc1cc(OC)c(O)cc1 | MOL002494 | 6-Gingesulfonic acid | G105 |
| C(CC(=O)C[C@H](CCCCC)O)c1cc(c(cc1)O)OC | MOL002467 | 6-Gingerol | G106 |
| C(c1cc(c(cc1)O)OC)CC(=O)/C=C(\O)/CCCCC | MOL002488 | 6-Gingerdione | G107 |
| c1(c(cc(cc1)CC[C@@H](C[C@@H](CCCCC)OC(=O)C)O)OC)O | MOL002492 | 6-Gingediol-5-acetate | G108 |
| c1(c(cc(cc1)CC[C@@H](C[C@@H](CCCCC)O)OC(=O)C)OC)O | MOL002491 | 6-Gingediol-3-acetate | G109 |
| C(C[C@@H](C[C@@H](CCCCC)O)O)c1cc(c(cc1)O)OC | MOL002493 | 6-Gingediol | G110 |
| c1(c(cc(cc1)CC[C@@H](C[C@@H](CCCCC)OC(=O)C)OC(=O)C)OC)O | MOL002490 | 6-Gingediacetate | G111 |
| C1(=CC[C@]2([C@@H]1C2)C(C)C)C | MOL001055 | 5-isopropyl-2-methylclo [3.1.0] Hec-2-ene | G112 |
| C(CC(=O)C[C@@H](CCc1ccc(cc1)O)O)c1cc(c(cc1)O)OC | MOL002487 | 5-Hydroxy-7-(4-hydroxyphenyl)-1-(4-hydroxy-3-methoxyphenyl)-3-heptanone | G113 |
| C(CC(=O)C[C@H](CCC)O)c1cc(c(cc1)O)OC | MOL002486 | 4-Gingerol | G114 |
| C1(=O)C=C[C@H](CC1)[C@@H](CCC=C(C)C)C | MOL002485 | 4-(1,5-Dimethylhex-4-enyl)cyclohex-2-enone | G115 |
| [C@@H]1(CCC=CC1)O | MOL002175 | 3-Cyclohexen-1-ol | G116 |
| C(=O)(CCCCCCC)C | MOL000918 | 2-Nonanone | G117 |
| C(=C(C)C)CCCC | MOL002481 | 2-Methylhept-2-ene | G118 |
| O1[C@@H](CC[C@@]1(C=C)C)C(O)(C)C | MOL001719 | 2-[(2S,5R)-5-Ethenyl-5-methyloxolan-2-yl]propan-2-ol | G119 |
| CC(C)(O)[C@H]1C[C@H]([C@](CC1)(C)C=C)C(=C)C | MOL000034 | 2-[(1R,3S,4S)-3-Isopropenyl-4-methyl-4-vinylcyclohexyl]propan-2-ol | G120 |
| c1c2nc(cc1)CCCCCCCCC2 | MOL002476 | 2,6-Nonamethylene pyridine | G121 |
| OC[C@H](O)COC(=O)CCCCCCC/C=C\C/C=C\CCCCC | MOL002464 | 1-Monolinolein | G122 |
| [C@@]12(OC([C@H](CC1)CC2)(C)C)C | MOL000122 | 1,8-Cineole | G123 |
| C(CCCC(=O)CCCCC)c1cc(c(cc1)O)OC | MOL002483 | 1-(4-Hydroxy-3-methoxyphenyl)decan-5-one | G124 |
| [C@H](CCCC(=C)C)(CCOC(=O)C)C | MOL002489 | [(3S)-3,7-Dimethyloct-7-enyl]acetate | G125 |
| [C@H](CCC=C(C)C)(CCOC(=O)C)C | MOL002455 | [(3S)-3,7-Dimethyloct-6-enyl]acetate | G126 |
| [C@H](CCOC(=O)CCC)(CCC=C(C)C)C | MOL002456 | [(3R)-3,7-Dimethyloct-6-enyl]butanoate | G127 |
| O1[C@@H]([C@H](O)[C@H](O)[C@@H](O)[C@@H]1OC[C@H](O)COC(=O)CCCCCCC/C=C\C/C=C\CCCCC)CO[C@H]1O[C@@H]([C@H](O)[C@H](O)[C@H]1O)CO | MOL002463 | [(2S)-2-Hydroxy-3-[(2R,3R,4S,5R,6R)-3,4,5-trihydroxy-6-[[(2S,3R,4S,5R,6R)-3,4,5-trihydroxy-6-(hydroxymethyl)tetrahydropyran-2-yl]oxymethyl]tetrahydropyran-2-yl]oxy-propyl] (9Z,12Z)-octadeca-9,12-dienoate | G128 |
| C/C(=C\[C@@H]1[C@H](C1(C)C)C(=O)O[C@@H]1C(=C(C(=O)C1)C/C=C/C)C)/C(=O)OC | MOL002501 | [(1S)-3-[(E)-But-2-enyl]-2-methyl-4-oxo-1-cyclopent-2-enyl] (1R,3R)-3-[(E)-3-methoxy-2-methyl-3-oxoprop-1-enyl]-2,2-dimethylcyclopropane-1-carboxylate | G129 |
| C(=O)(C)O[C@H]1[C@H](CC[C@H](C1)C)C(C)C | MOL002471 | [(1R,2R,5R)-2-Isopropyl-5-methyl-cyclohexyl] acetate | G130 |
| [C@]12([C@H](C([C@@H](C1)CC2)(C)C)OC(=O)C)C | MOL002520 | .beta.-Fenchyl acetate, exo- | G131 |
| C(/C=C(/C)\CC/C=C(/C)\CCC=C(C)C)O | MOL000247 | (Z,Z)-Farnesol | G132 |
| [C@@H]1(C(C)C)CC=C(C=C1)C | MOL000200 | (S)-(+)-alpha-Phellandrene | G133 |
| [C@@](CCC=C(C)C)(C=C)(C)O | MOL000198 | (R)-Linalool | G134 |
| CC(C)(O)[C@@H]1CC=C(CC1)C | MOL000118 | (L)-alpha-Terpineol | G135 |
| c1(cc(c(cc1)O)OC)CCC(=O)C[C@H](CCCCCCC)O | MOL002497 | (8)-Gingerol | G136 |
| C1(=CC[C@H](CC1)C(C)C)C=O | MOL001210 | (4S)-4-Isopropylcyclohexene-1 | G137 |
| [C@H](CCC)(C=O)C | MOL002479 | (2S)-2-Methylpentanal | G138 |
| C(CCCC)[C@@H](C)O | MOL002478 | (2R)-Heptan-2-ol | G139 |
| [C@@H](CCC=C(C)C)(C=O)C | MOL002477 | (2R)-2,6-Dimethylhept-5-enal | G140 |
| [C@]12([C@@H](C1)C(=C)CC2)C(C)C | MOL000268 | (1S,5S)-1-Isopropyl-4-methylenebicyclo[3.1.0]hexane | G141 |
| [C@H]1(CCC(=C)[C@@H]2CCC(=C[C@@H]12)C)C(C)C | MOL000489 | (1S,4aR,8aR)-1-Isopropyl-7-methyl-4-methylene-2,3,4a,5,6,8a-hexahydro-1H-naphthalene | G142 |
| C1=C([C@H]2C[C@@H](C1)[C@]2(C)CCC=C(C)C)C | MOL001201 | (1R,5R,7S)-4,7-Dimethyl-7-(4-methylpent-3-enyl)bicyclo[3.1.1]hept-3-ene | G143 |
| [C@]12([C@@H](C[C@@H](CC1)C2(C)C)O)C | MOL000018 | (+_-)-Isoborneol | G144 |
| CC([C@H]1C=CC(=C)CC1)C | MOL002028 | (+)-beta-Phellandrene | G145 |
| c1(ccc(cc1)[C@@H](C)CCC=C(C)C)C | MOL000890 | (+)-alpha-Curcumene | G146 |
| [C@@]123[C@@](CC[C@H]1C)(C(=O)CC[C@@H](C2)C(=C)C)O3 | MOL002450 | (+)-1,5-Epoxy-nor-ketoguaia-11-ene | G147 |
| [C@H]12C([C@H](C1)CCC2=C)(C)C | MOL000126 | (-)-Nopinene | G148 |
| [C@H]12C(C)(C)C(=C)[C@H](CC1)C2 | MOL002453 | (-)-Comphene | G149 |
| [C@H](CCC=C(C)C)(CC=O)C | MOL000774 | (-)-Citronellal | G150 |
| [C@]12(C([C@@H](C[C@@H]1O)CC2)(C)C)C | MOL000244 | ()-Borneol | G151 |
| [C@H](CCC=C(C)C)(C=C)C | MOL002482 | ()-beta-Citronellene | G152 |
| [C@@H]12C(=CC[C@@H](C1)C2(C)C)C | MOL000125 | (-)-alpha-Pinene | G153 |
| [C@]123[C@H](C([C@@H](C1)C(=CC3)C)(C)C)CC[C@H]2C | MOL000612 | (-)-alpha-Cedrene | G154 |
| [C@@H]1([C@H](CCC=C(C)C)C)CCC(=C)C=C1 | MOL002452 | β-Sesquiphellandrene | G155 |
| C1(C/C=C(/CCC(=C)C/C=C/1)\C)(C)C | MOL002451 | β-Humulene | G156 |
| O1[C@]2(CC[C@@H](C1)C)O[C@@H]1[C@@H]([C@@H]2C)[C@@]2([C@@H](C1)[C@H]1[C@H](CC2)[C@@]2(C(=CC1)C[C@H](CC2)O)C)C | MOL002518 | Zingiberoside A3_qt | G157 |
| **Coptidis Rhizoma (huanglian)** | | | |
| c1c2c(c3c(c1)ccc(=O)o3)C[C@H](O2)C(OC(=O)/C(=C\C)/C)(C)C | MOL002905 | Zosimin | H1 |
| O1c2cc3C4=C(c5c(C=N4CCc3cc2OC1)cc1OCOc1c5)C | MOL002668 | Worenine | H2 |
| c1(cc(c(cc1)O)OC)C(=O)O | MOL000114 | vanillic acid | H3 |
| C1[C@H](C[C@@H]2[C@](C1)([C@@H]1[C@@H]([C@@H](C2)O)[C@@H]2[C@]([C@H](C1)O)([C@@H](CC2)[C@@H](C)CC[C@@H](C)[C@@H](C)C(=O)O)C)C)O | MOL002893 | Trihydroxybufosterocholanic acid | H4 |
| O1c2c3[C@@H](N(CCc3cc(OC)c2OC)C)Cc2cc(Oc3ccc(C[C@@H]4N(CCc5c4cc1c(OC)c5)C)cc3)c(OC)cc2 | MOL002343 | tetrandrine | H5 |
| c1(cc(c2c(c1)oc(c(c2=O)O)c1ccc(c(c1)O)O)O)O | MOL000098 | quercetin | H6 |
| c12c3c(c(cc1CCN([C@@H]2Cc1cc(Oc2ccc(C[C@@H]4c5cc(O3)c(cc5CCN4C)OC)cc2)c(cc1)O)C)OC)OC | MOL006972 | Pycnamine | H7 |
| c12c(cc(c(c1)O)OC)CN1([C@H](C2)c2c(CC1)cc(c(c2)O)OC)C | MOL002901 | phellodendrine | H8 |
| C(=O)(/C=C/c1ccc(cc1)O)O | MOL000771 | p-coumaric acid | H9 |
| c12c([C@H](c3c(C1=O)c(cc(c3)C)O)[C@H]1c3c(C(=O)c4c1cc(cc4O)CO)c(ccc3)O)cc(cc2O)O | MOL000762 | Palmidin A | H10 |
| C12=Cc3c(C=N2CCc2c1cc(c(c2)OC)OC)c(c(cc3)OC)OC | MOL000785 | palmatine | H11 |
| O1c2c3[C@@H](N(CCc3cc(OC)c2OC)C)Cc2ccc(Oc3cc(C[C@H]4N(CCc5c4cc1c(OC)c5)C)ccc3O)cc2 | MOL013012 | Oxyacanthine | H12 |
| C1CN([C@H]2c3c1cc(c(c3Oc1c(OC)cc3c(c1)[C@@H](N(CC3)C)Cc1ccc(Oc3c(ccc(C2)c3)O)cc1)O)OC)C | MOL002639 | Obamegine | H13 |
| O1[C@@]23[C@]4([C@@H]([C@@]5([C@@H](CC4=O)C(OC(=O)C=C5)(C)C)C)CC[C@]2([C@@H](OC(=O)[C@@H]13)c1ccoc1)C)C | MOL013352 | Obacunone | H14 |
| c1cc(co1)[C@H]1[C@]2([C@]3([C@@H](C(=O)O1)O3)[C@]1([C@H](CC2)[C@]([C@@H](CC1=O)C(O)(C)C)(/C=C/C(=O)O)C)C)C | MOL002637 | Obacunoic acid | H15 |
| c12c(cc3c(c1)OCO3)CCNC2=O | MOL002900 | Noroxyhydrastinine | H16 |
| O(c1cc(/C=C/C(=O)NCCc2ccc(O)cc2)ccc1O)C | MOL008647 | Moupinamide | H17 |
| C(=O)(c1cc(c(cc1)O)O)OC | MOL003503 | Methyl protocatechuate | H18 |
| [C@@H]12[C@@H]([C@@H]3[C@@H](CC[C@@]1(C)O)C(=C)C(=O)O3)[C@@](CC2)(O)C | MOL000622 | Magnograndiolide | H19 |
| Oc1c2c3[C@@H](N(CCc3cc1OC)(C)C)Cc1c2c(O)c(OC)cc1 | MOL002891 | magnoflorine | H20 |
| O1[C@@]23[C@]4([C@@H]([C@]56[C@@H](CC4=O)C(O[C@H]5CC(=O)OC6)(C)C)CC[C@]2([C@@H](OC(=O)[C@@H]13)c1ccoc1)C)C | MOL003959 | limonin | H21 |
| C1(=O)C[C@@]2([C@H]3[C@@H](O1)[C@@H]([C@@]1([C@H]4[C@@]3([C@@H](C[C@H]2C(=O)C)OC(=O)[C@@H]4OC(=O)C)CO1)C(=O)OC)OC(=O)C)C | MOL002329 | Javanicin | H22 |
| C12=Cc3c(C=N2CCc2c1cc(c(c2)O)OC)c(c(cc3)OC)OC | MOL000789 | jatrorrizine | H23 |
| O(c1c2c[n+]3CCc4c(c3cc2ccc1OC)cc(OC)c(O)c4)C | 72323 | Jatrorrhizine | H24 |
| Oc1cc(C=O)ccc1OC | MOL001867 | Isovanillin | H25 |
| c1(ccc(cc1O)CCO)O | MOL000141 | hydroxytyrosol | H26 |
| O([C@@H]1C[C@@](O)(C[C@@H](O)[C@H]1O)C(=O)O)C(=O)/C=C/c1cc(O)c(O)cc1 | MOL001955 | Heriguard | H27 |
| c12c(CCN3=C1C=c1c(=C3)c3OCOc3cc1)cc(c(c2)OC)O | MOL002898 | groenlandicine | H28 |
| c1(c(cc(cc1)O)C(=O)O)O | MOL003178 | GENOP | H29 |
| c1(cc(c(cc1)O)OC)/C=C/C(=O)O | MOL000360 | FER | H30 |
| o1c2nc3c(c(OC)c2cc1)cccc3OC | MOL002664 | Fagarine | H31 |
| c1(cc(c(cc1)O)O)/C=C/C(=O)OCC | MOL002902 | Ethyl caffeate | H32 |
| O1c2c3=CN4=C(c5c(CC4)cc(OC)c(OC)c5)C=c3ccc2OC1 | MOL002897 | epiberberine | H33 |
| c1c(c2c(c(c1)O)oc(cc2=O)CCc1ccccc1)O | MOL002895 | DPEC | H34 |
| O1[C@@H]([C@@H](O)[C@H](O)[C@@H](O)[C@@H]1OCCc1cc(O)c(O)cc1)CO | 5316821 | Dopaol beta-D-glucoside | H35 |
| c1(c(cc(cc1)C[C@H](C(=O)O)O)O)O | MOL007134 | danshensu | H36 |
| c12c(cc(c(c1)OC)OC)C(=O)NCC2 | MOL002896 | Corydaldine | H37 |
| C1[C@H](C[C@@]2([C@@](C1)([C@@H]1[C@@H](CC2)[C@@]2([C@](CC1)([C@@H](CC2)C1=CC(=O)OC1)C)O)C=O)O)O | MOL002907 | Corchoroside A_qt | H38 |
| C1[C@@H](C[C@@]2([C@@](C1)([C@@H]1[C@@H](CC2)[C@@]2([C@](CC1)([C@H](CC2)C1=CC(=O)OC1)C)O)C=O)O)O[C@@H]1O[C@@H]([C@@H]([C@H](C1)O)O)C | MOL002906 | Corchoroside A | H39 |
| O1c2cc3C4=N(CCc3cc2OC1)C=c1c(=C4)ccc2OCOc12 | MOL001458 | coptisine | H40 |
| O1[C@@H](C(OC(=O)C(=CC)C)(C)C)Cc2c1ccc1c2oc(=O)cc1 | 53399217 | columbianadin | H41 |
| O(c1c2=CN3=C(c4c(CC3)cc(OC)c(O)c4)C=c2ccc1OC)C | MOL001457 | columbamine | H42 |
| Oc1ccc(cc1OC)[C@@H]1OC[C@H](Cc2ccc(c(c2)OC)O)[C@H]1CO | MOL001845 | clemastanin B_qt | H43 |
| C(=C\c1cc(c(cc1)OC)OC)\C(=O)O | MOL004095 | Cinnamic acid, 3,4-dimethoxy- (8CI) | H44 |
| OC1(CC(C(O)C(O)C1)C)C(=O)O.Oc1cc(ccc1O)C=CC | 53399195 | chlorogenic acid | H45 |
| c12c3c(cc4c(c3)OCO4)CCn1c(=O)c1c(c2)ccc(c1OC)OC | MOL002904 | Berlambine | H46 |
| O1c2cc3CCN4=Cc5c(C=C4c3cc2OC1)ccc(OC)c5O | MOL002894 | berberrubine | H47 |
| O1c2cc3CCN4=C(c3cc2OC1)C=c1c(=C4)c(OC)c(OC)cc1 | MOL001454 | berberine | H48 |
| COc1c(O)ccc(c1)/C=C/C(=O)O[C@@H]1C[C@]([C@@H]2[C@H]1C=CO[C@H]2O)(C)O | MOL000779 | 6-O-E-Feruloylajugol_qt | H49 |
| COc1c(O)ccc(c1)/C=C/C(=O)O[C@H]1C[C@]([C@@H]2[C@H]1C=CO[C@H]2O[C@@H]1O[C@@H]([C@H]([C@@H]([C@H]1O)O)O)CO)(C)O | MOL000778 | 6-O-E-Feruloylajugol | H50 |
| O1[C@@H]([C@@H](O)[C@H](O)[C@@H](O)[C@@H]1Oc1cc(c(O)cc1)C(=O)O)CO | 10914066 | 5-(beta-D-Glucopyranosyloxy)-2-hydroxybenzoic acid | H51 |
| Oc1c(CCC(=O)O)ccc(O)c1O | 5322049 | 3-(2,3,4-Trihydroxyphenyl)propanoic acid | H52 |
| c1ccc2c(c1)C(=O)[C@]1([C@](C2=O)(CC=C(C)C)O1)C(=O)OC | MOL002890 | 2-Carboxymethyl-3-prenyl-2,3-epoxy-1,4-naphthoquinone | H53 |
| c12c(c(c(cc1)OC)OC)CN1[C@H](C2)c2c(CC1)cc1c(c2)OCO1 | MOL002903 | (R)-Canadine | H54 |
| O1[C@@H]([C@@H](O)[C@@H](O)[C@@H](O)[C@@H]1OCCc1cc(O)c(O)cc1)CO | 6915839 | (2R,3R,4R,5S,6R)-2-[2-(3,4-Dihydroxyphenyl)ethoxy]-6-(hydroxymethyl)oxane-3,4,5-triol | H55 |
